# A troglomorphic spider from Java (Araneae, Ctenidae, *Amauropelma*)

**DOI:** 10.3897/zookeys.163.2265

**Published:** 2012-01-09

**Authors:** Jeremy Miller, Cahyo Rahmadi

**Affiliations:** 1 Department of Entomology, Netherlands Centre for Biodiversity Naturalis, Postbus 9517 2300RA Leiden, The Netherlands; 2 Department of Entomology, California Academy of Sciences, 55 Music Concourse Drive, Golden Gate Park, San Francisco, CA 94118, USA; 3 Museum Zoologicum Bogoriense, Research Center for Biology, Indonesian Institute of Sciences, Jalan Raya Bogor km 46, Cibinong, 16911 West Java, Indonesia

**Keywords:** conservation, DNA barcode, Indonesia, Jonggrangan Limestone, troglobite

## Abstract

A new troglomorphic spider from caves in Central Java, Indonesia, is described and placed in the ctenid genus *Amauropelma* Raven, Stumkat & Gray, until now containing only species from Queensland, Australia. Only juveniles and mature females of the new species are known. We give our reasons for placing the new species in *Amauropelma*, discuss conflicting characters, and make predictions about the morphology of the as yet undiscovered male that will test our taxonomic hypothesis. The description includes DNA barcode sequence data.

## Introduction

We describe a new troglobitic spider taken from caves in Central Java, Indonesia. The species is a ctenid tentatively placed in the genus *Amauropelma*, which was established to accommodate 16 new species from Queensland, Australia by Raven et al. in 2001. Ours is the first new *Amauropelma* species to be proposed since Raven et al.’s original description. We are writing this paper as the description of a single species known from one sex because it is a troglobite (and therefore of potential conservation interest) from a taxon for which good comparative descriptive data are available. Although few specimens have been collected, many more specimens have been observed and not collected out of prudent concern for the population. However, all of these observations were of either juveniles or mature females. Repeated attempts to target males have so far failed.

## Methods

Characters described mostly follow Raven et al. (2001) to facilitate comparison with known species. Observations of vulva structures were made based on a dissected epigynum cleared in methyl salicylate (Holm 1962), positioned using a temporary slide mount (Coddington 1983), and viewed through a Leica DM2500 compound microscope. Other observations were made based on specimens in alcohol viewed through a Leica M165 C stereoscope. Photographs were made using a Nikon DS-Ri1 driven by NIS Elements software and mounted on either the DM2500 microscope or the M165 C stereoscope. Images from multiple focal planes were combined and edited in Auto-Montage software version 5.03. Additional processing of some images to adjust color, brightness, and contrast, and remove blemishes was performed using Adobe Photoshop CS5. Tarsal organ position expressed as a ratio of the distance from the proximal margin of the tarsus to the tarsal organ divided by the total length of the tarsus. All measurements in millimeters. Abbreviations given in Table 1.

We used the Pensoft IPT Data Hosting Center to expose specimen occurrence records to the Global Biodiversity Information Facility (GBIF; http://data.gbif.org/welcome.htm). A KML (Keyhole Markup Language) file for viewing these same specimen occurrence records interactively in Google Earth (http://earth.google.com/) is available as electronic appendix A. In accordance with Pensoft’s practice of semantic markup and publishing, the species described herein has been registered on ZooBank (http://zoobank.org/) and a species page has been submitted to the Encyclopedia of Life (http://www.eol.org/) and the wiki species-id (http://species-id.net/wiki/).

658 bases of cytochrome oxidase I were sequenced by the NCB Naturalis DNA barcoding facility using the following primers: LCO1490 (5’-GGTCAACAAATCATAAAGATATTGG-3’) and HCO2198 (5’-TAAACTTCAGGGTGACCAAAAAATCA-3’) (Folmer et al. 1994). Chromatogram data are available as electronic appendix B.

**Table 1.** List of abbreviations used in the text and figures.

**Spinnerets and somatic morphology:**	
ALE	anterior lateral eye
ALS	anterior lateral spinneret
AME	anterior median eye
AT	anal tubercle
CD	copulatory duct
ET	epigynal tooth
fe	femur
me	metatarsus
p	prolateral
pa	patella
PLE	posterior lateral eye
PLS	posterior lateral spinneret
PME	posterior median eye
PMS	posterior median spinneret
r	retrolateral
S	spermatheca
ti	tibia
v	ventral
	
**Institutional abbreviations:**	
MZB	Museum Zoologicum Bogoriense, Bogor
RMNH	Netherlands Centre for Biodiversity Naturalis, Leiden

## Taxonomy

### Amauropelma

Raven, Stumkat & Gray, 2001

http://species-id.net/wiki/Amauropelma

#### Type species.

*Amauropelma trueloves* Raven & Stumkat, 2001

#### Addendum to diagnosis.

Tarsal organ position ranges from 0.125–0.77. Tarsi with or without adpressed trichobothria. Epigynum with soft or sclerotized lateral teeth. Tracheal spiracle distinct or indistinct. Otherwise, as in Raven et al. (2001).

### Amauropelma matakecil

Miller & Rahmadi sp. n.

urn:lsid:zoobank.org:act:180E7280-7D8D-4884-81FC-C8BE75FDD361

http://species-id.net/wiki/Amauropelma_matakecil

Figs 1 [Fig F2] 13

#### Material examined.

Holotype: Indonesia, Central Java, Purworejo, Kaligesing, Tlogoguo Village, Somoroto: Gua Anjani [Anjani Cave], 7.73156°S, 110.11567°E, 672 m asl., 23 March 2009 (MZB.Aran.500, S. Harjanto), 1 #f.

Paratypes: Indonesia, Central Java, Purworejo, Kaligesing, Donorejo Village, Katerban: Gua Seplawan [Seplawan Cave], 7.7726°S, 110.111°E, 23 April 2010 (MZB.Aran.501, S. Harjanto and C. Rahmadi), 1 #f; Indonesia, Central Java, Purworejo, Kaligesing, Donorejo Village, Katerban: Gua Nguwik [Nguwik Cave], 7.76907°S, 110.10334°E, 764 m asl., 9 May 2008 (RMNH.ARA.12434, S. Harjanto), 1 #f.

Additional material examined: Indonesia, Central Java, Purworejo, Kaligesing, Tlogoguo Village, Somoroto: Gua Anjani [Anjani Cave], 7.73156°S, 110.11567°E, 672 m asl., 23 April 2010 (RMNH.ARA.12436, S. Harjanto and C. Rahmadi), 2 juveniles; Gua Anjani [Anjani Cave], 7.73156°S, 110.11567°E, 672 m asl., 23 March 2009 (MZB.Aran.502, S. Harjanto), 1 juvenile.

#### Etymology.

The specific name is an adjective derived from "mata" meaning eyes and "kecil" meaning small from Bahasa Indonesia referring to the small eyes of the species. *Pronunciation note*: the letter “c” in Bahasa is pronounced like “ch” in English.

#### Diagnosis

**Figures 1–6.**
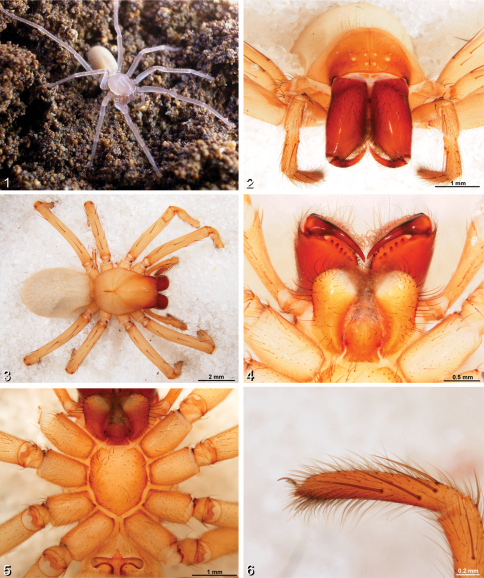
*Amauropelma matakecil* sp. n. **1** female habitus **2–6** habitus of female holotype (MZB.Aran.500) **1** Portrait of live specimen in natural habitat from Gua Nguwik, Central Java (Photo S. Harjanto) **2** Anterior view **3** Dorsal view **4** Ventral view showing labium, endites, and chelicerae **5** Ventral view showing sternum, coxae, and trochanters **6** Left pedipalpal, retrolateral view.

**Figures 7–12. F2:**
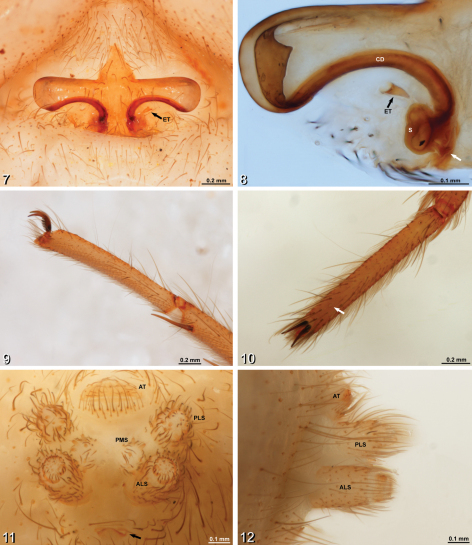
*Amauropelma matakecil* sp. n., female holotype (MZB.Aran.500) **7** Epigynum, ventral view. Note that the right epigynal tooth has broken off leaving a round hole; the tooth itself is lying unattached near the epigastric furrow **8** Vulva, dorsal view, left side, cleared, white arrow indicates fertilization duct **9** Right tarsus, leg I, prolateral view **10** Left tarsus, leg I, dorsal view, arrow indicates tarsal organ **11** Spinnerets, anal tubercle, and tracheal spiracle, posterior view, arrow indicates tracheal spiracle **12** Spinnerets, lateral view. ALS, anterior lateral spinneret; AT, anal tubercle; CD, copulatory duct; ET, epigynal tooth; PLS, posterior lateral spinneret; PMS, posterior median spinneret; S, spermatheca.

**.** Distinguished from other *Amauropelma* species by having more cheliceral teeth (4 promargin and 7 retromargin teeth, Fig. 4; other described species for which data were recorded range between 1–4 promargin and 4–6 retromargin teeth); by the relatively proximal position of the tarsal organs (Fig. 10); by the sclerotized epigynal teeth that do not conduct the copulatory ducts (Fig. 7; other *Amauropelma* have soft teeth containing the copulatory ducts); and by the shape of the epigynum, which has the lateral wings more long and narrow than other species (Fig. 7). Further distinguished from other *Amauropelma* species except *Amauropelma leo* Raven & Sumkat, 2001 by having small eyes (Fig. 2; large in other species except *Amauropelma undara*, which is a blind troglobite); distinguished from *Amauropelma leo* by the pale, troglomorphic color (Figs 1, 3); *Amauropelma leo* is a rainforest species and is not troglomorphic.

#### Description.

Female (holotype, MZB.Aran.500): Carapace 3.40 long, 2.20 wide. Abdomen 4.12, long 2.64 wide. Total length 7.7. Carapace with fine setae. Fovea a narrow groove. Chilum divided (Fig. 2). Endites slightly converging, labium longer than wide (Fig. 4). *Color*. Overall pale, chelicerae and ocular region darker (Figs 1, 3). *Eyes*. Vestigial, eye rows recurved forming 2.4.2 pattern, ALE>AME=PME>PLE, AME on small common tubercle, ALE and PME form slightly procurved row, PME closer together than PME-ALE, PME slightly more widely spaced than AME, eye group = 0.40 of carapace width (Figs 2, 3). *Chelicerae*. Large, partially porrect with lateral boss. Retromargin with 5 large distal and 2 small proximal teeth; promargin with 4 teeth, the third (counting proximally from the base of the fang) is the smallest (Fig. 4). *Pedipalp*. Tarsal claw with series of basal teeth. With two large ventral distal setae (ca. Silva Dávila 2003: fig. 28d). *Legs*. Formula 4123. Paired tarsal claws with series of basal teeth, claw tufts present, weak scopula present on tarsi and metatarsi I and II (Fig. 9). Retrocoxal hymen present on leg I. Trochanters deeply notched (Fig. 5). Tarsal organs slightly raised, dome-like, more distal on legs I and II than III and IV (I: 0.77. II: 0.73. III: 0.66. IV: 0.67). Macrosetae: I: fe p1d3; pa 0; ti v2.2.2.2.2; me v3.3.3; ta 0. II: fe d3r1; pa 0; ti v2.2.2.2.2; me v3.3.3; ta 0. III: fe p4d3r4; pa p1r1; ti v2.2.2p1.1d1.1 r1.1; me v2.2.2p1.1.2r1.1.2; ta 0. IV: fe p3d3r1; pa r1; ti v2.2.2p1.1d1.1r1.1; me v1.1.1.1.2p1.1.1d0.1.2r1.1.1. *Spinnerets*. Ecribellate, colulus absent, lateral spinnerets cylindrical with short apical segment, ALS separated by about their width, PLS and PMS with a number of large, conspicuous spigots (Figs 11, 12). *Epigynum*. Sclerotized plate with long, narrow lateral wings with concave posterior margins. Epigynal teeth sclerotized, arise posterior to lateral wings (Fig. 7). Copulatory openings on dorsal surface near lateral margins of wings, follow posterior margin of wings to reniform spermathecae (Fig. 8).

**Figure 13.**
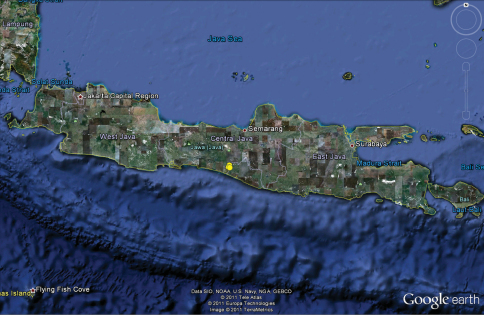
Map of Java, Indonesia, showing records of *Amauropelma matakecil* sp. n. as yellow circles in Central Java. Base map source: Google Earth.

#### Natural History.

In Seplawan Cave, *Amauropelma matakecil* was found on the cave floor hiding under crevices in dry mud.

#### Distribution.

*Amauropelma matakecil* is known only from three caves in the Jonggrangan Limestone, part of the Menoreh Hills in the District of Kaligesing, Purworejo Regency, Central Java, near the border with Yogyakarta Province (Fig. 13). The Jonggrangan Limestone is located from 574–878 m above sea level (Bemellen 1949). This karst formation is a fossil reef with thicknesses up to 200 m at the southern margin of the Jonggrangan Plateu (Bemellen 1949). The formation dates from the Middle to Late Miocene (Sulistyaningrum and Rahardjo 2010). Karst makes up a very small area of the Menoreh hills, about 15 km^2^. The nearest neighboring limestone formations are the Gombong Selatan (about 72 km to the west) and the Gunung Sewu Karst (about 42 km to the east).

#### Remarks.

The cave spider fauna of Java is not well known. The only other spider documented from a cave in Java that we are aware of is *Althephus javanensis* Deeleman-Reinhold, 1995 (Ochyroceratidae). This species is not strongly troglomorphic, exhibiting neither eye reduction nor reduced pigmentation, although legs in specimens from caves are considerably longer than in specimens from the surface. As reported by Rahmadi (2011), *Amauropelma matakecil* is the most remarkable cave spider so far known from Java due to its large size, reduced eyes, and potential conservation importance. Karst formations in Java are highly threatened by human activities such as limestone mining and habitat conversion.

#### DNA Barcode.

AACGTTATATTTAATATTTGGAGCTTGATCTGC TATAATAGGAACGGCTATAAGAATATTAATTCGAATAGAGTTAGGA CATTCTGGAAGATTATTAAGTAATGATCATTTGTATAATGTGATTGT TACTGCTCATGCATTTGTTATAATTTTTTTTATGGTGATGCCAATTT TAATTGGAGGTTTTGGAAATTGATTAGTTCCTTTAATATTAGGAGCTC CGGATATATCGTTTCCTCGAATAAATAATTTGTCTTTTTGATTGTTAC CTCCTTCTTTGTTTTTGTTGTTTATATCTTCTATAGTTGAAATGG GAGTAGGAGCTGGATGAACTATTTATCCCCCTTTAGCTTCTAGAATTG GTCATGTGGGAAGATCTATGGATTTTGCTATTTTTTCTTTACATT TAGCTGGAGCTTCTTCTATTATAGGGGCGGTAAATTTTATTTCTAC GATTGTAAATATACGTTTATTAGGAATAAGAATAGAAAGGGTTCCTT TATTTGTGTGATCTGTATTTATTACTGCTGTTTTATTATTATTATCTT TACCTGTTTTAGCGGGAGCTATTACTATGTTATTGACGGATCGAAATTT TAATACTTCTTTTTTTGACCCTGCAGGGGGAGGGGATCCTATTT TATTTCAACATTTGTTT (MZB.Aran.501, GenBank accession number JQ277219).

Among identified spiders accessible at the time of writing (October 2011) through the NCBI database (http://blast.ncbi.nlm.nih.gov/Blast.cgi), *Amauropelma matakecil* blasts most closely with the pisaurid genus *Dolomedes* Latreille, 1804. This despite the presence in GenBank of the homologous locus for several ctenid spiders (e.g., Crews and Gillespie 2010). However, its closest matches are several still unidentified spiders in the International Barcode of Life (iBOL) database.

## Discussion

The species described here appears to fit best in the genus *Amauropelma* based on several characters including the eye arrangement (Fig. 2), the presence of only the superior tarsal claws (no inferior tarsal claw; Fig. 9), the leg spination pattern, the presence of two ventral distal macrosetae on the female pedipalp (ca. Silva Dávila 2003: fig. 28d), and lateral wings and posterior teeth on the epigynum (Fig. 7). However, *Amauropelma matakecil* exhibits characteristics that are not typical of *Amauropelma* and none of the above characters are unique to *Amauropelma*.

The form of the epigynum is also similar to the genera *Thoriosa* Simon (from West Africa and nearby Atlantic islands) and some *Trogloctenus* Lessert (from Congo and Réunion). Silva Dávila’s (2003) phylogenetic analysis placed *Thoriosa* close to *Amauropelma* and an incertae sedis species from Lombok Island, Indonesia; *Trogloctenus* was not included in that analysis due to a lack of non-type material in collections. *Amauropelma* including our new species differs from *Thoriosa* and *Trogloctenus* by the eye arrangement. *Thoriosa* has the median ocular area wider posteriorly than anteriorly (Benoit 1976: figs 1, 4); in *Amauropelma* including our new species, the median ocular area is as wide anteriorly as posteriorly. In the type species of *Trogloctenus*, the clypeus is about seven AME diameters (Benoit 1976: fig. 12); in *Amauropelma*, the clypeus ranges from less than one to about two AME diameters. A second species of *Trogloctenus* has no eyes so this character is inapplicable, but in this species the lateral wings of the epigynum are not so pronounced and posterior teeth are apparently absent (Ledoux 2004: fig. 9B). The loss of the inferior tarsal claw, the presence of two ventral distal macrosetae on the female pedipalp (ca. Silva Dávila 2003: fig. 28d) and the leg spination pattern are all found in multiple ctenid genera including *Thoriosa*.

There are also some characteristics that conflict with *Amauropelma*. The epigynal teeth of the new species are hard rather than soft. The copulatory openings appear to be associated with the anteriomesal part of the lateral wings of the epigynum rather than with the epigynal teeth (Fig. 8). The claw tufts are less dense than in other *Amauropelma* species (Fig. 9). The position of the tarsal organs is much more distal than that reported for other *Amauropelma* species (Fig. 10). Note that the tarsal organ of *Janusia* Gray is described as subdistal and distal to trichobothria (Gray 1973; see below). The tracheal spiracle is small but easy to see because of a narrow sclerotized margin (Fig. 11; Raven et al. 2001 reported the tracheal spiracle of *Amauropelma* indistinct). Raven et al. (2001) described the labium as longer than wide. Based on illustrations (Raven et al. 2001: fig. 5C, 21G), this condition is amplified in the new species (labium length 1.3 times the width; Fig. 4). Adpressed trichobothria were not observed on the tarsi of our new species, as reported by Raven et al. for *Amauropelma* (e.g., Raven et al. 2001: fig. 3f) but are apparently present on the tibiae. It seems clear that there are several ctenid lineages closely related to *Amauropelma* that would benefit from revision and more extensive illustration.

One other troglobitic *Amauropelma* is known. *Amauropelma undara* Raven & Stumkat from lava tubes in Queensland is completely blind, in contrast to our new species which has vestigial eyes. Another ctenid known from caves that shares characteristics with our new species is the genus *Janusia* (see Raven et al. 2001). This genus contains only one described species from Western Australia but the existence of possibly congeneric undescribed species has been reported (Gray 1973; Raven et al. 2001). Our new species can be separated from *Janusia muiri* Gray in part by the presence of a small inferior tarsal claw in *Janusia* (no inferior tarsal claw in *Amauropelma*) and by the presence of only three teeth on the superior tarsal claws (ca. 7 in our new species; Fig. 7).

Based on the characteristics of other *Amauropelma* species, we predict that the male when discovered will be found to exhibit no tibial crack on the legs, will have retrolateral processes on the palpal patella and tibia, will have an apically coniform cymbium without a dorsal scopula, will have a palpus with a cup-shapped median apophysis, a hyaline conductor, an embolus in the form of a large hook-shaped plate, and other anatomical details in common with known *Amauropelma* species. If these predictions are not borne out with the eventual discovery of the male, the generic position of this species may have to be reconsidered. The male of *Janusia* has not been described, but based on a broken embolus extracted from the reproductive tract of a female, the embolus is thin and coil-like (Gray 1973).

## Supplementary Material

XML Treatment for Amauropelma

XML Treatment for Amauropelma matakecil

## Appendix A

Specimen records of *Amauropelma matakecil*. (doi: 10.3897/zookeys.163.2265.app1) File format: KML (Keyhole Markup Language) version 2.1 for GoogleEarth.

**Explanation note:** The KML file can be opened using GoogleEarth (http://earth.google.com/) to display an interactive map showing the specimen occurrence data for *Amauropelma matakecil*.

Click on placemarks to reveal specimen data and a hyperlink to the species page on the Encyclopedia of Life (http://www.eol.org/).

**Copyright notice:** This dataset is made available under the Open Database License (http://opendatacommons.org/licenses/odbl/1.0/). The Open Database License (ODbL) is a license agreement intended to allow users to freely share, modify, and use this Dataset while maintaining this same freedom for others, provided that the original source and author(s) are credited.

## Appendix B

DNA barcode. (doi: 10.3897/zookeys.163.2265.app2) File format: SPF (Sequencher Project File).

**Explanation note:** Chromatograms for the DNA barcode sequence of *Amauropelma matakecil*.

**Copyright notice:** This dataset is made available under the Open Database License (http://opendatacommons.org/licenses/odbl/1.0/). The Open Database License (ODbL) is a license agreement intended to allow users to freely share, modify, and use this Dataset while maintaining this same freedom for others, provided that the original source and author(s) are credited.
